# Factors Associated with Cancer-Related Pain Requiring High-Dose Opioid Use in Palliative Cancer Patients

**DOI:** 10.1089/pmr.2021.0037

**Published:** 2021-09-15

**Authors:** Hidetoshi Sumimoto, Komaki Hayashi, Yuri Kimura, Akihito Nishikawa, Seiko Hattori, Chiaki Hasegawa, Hiroaki Morii, Koji Teramoto, Sachiyo Morita, Yataro Daigo

**Affiliations:** ^1^ Department of Medical Oncology, Shiga University of Medical Science Hospital, Otsu, Shiga, Japan.; ^2^ Cancer Center, Shiga University of Medical Science Hospital, Otsu, Shiga, Japan.; ^3^ Palliative Care Center, and Shiga University of Medical Science Hospital, Otsu, Shiga, Japan.; ^4^ Department of Pharmacy, Shiga University of Medical Science Hospital, Otsu, Shiga, Japan.

**Keywords:** cancer-related pain, intractable cancer pain, high-dose opioid

## Abstract

***Background:*** There are no universal tools to predict the necessity of high-dose opioid use for cancer-related pain. Early recognition and interventions for intractable cancer pain could minimize the distress of palliative patients.

***Objective:*** We sought to identify the clinical factors associated with high-dose opioid use in advanced cancer patients to recognize palliative patients who would develop intractable cancer pain, as early as possible.

***Setting/Subjects:*** Among 385 in-hospital cancer patients from April 1, 2014 to July 31, 2019, who were referred to the palliative care team for cancer-related pain, clinical factors significantly correlated to high-dose opioid use were retrospectively analyzed.

***Measurements:*** We conducted a multiple logistic regression analysis to identify variables significantly related to high-dose opioid use (>120 mg/day oral morphine equivalent dose).

***Results:*** Independent factors of high-dose opioid use included younger age (odds ratio [OR] 0.965, 95% confidence interval [CI] 0.944–0.986, *p* = 0.001), respiratory cancers (OR 1.882, 95% CI 1.069–3.312, *p* < 0.001), and opioid switch (OR 2.869, 95% CI 1.497–5.497, *p* = 0.001). The percentage of correct classifications of the regression equation was 86.9%.

***Conclusions:*** Younger age, respiratory cancers, and opioid switch were related to high-dose opioid use. Our findings may help palliative caregivers to deal with intractable cancer pain in palliative patients, and thus relieve their distress.

## Introduction

Cancer-related pain is one of the most frequent and distressing symptoms experienced by palliative patients.^1^ Although most of the cancer-related pain could be well controlled by following the World Health Organization guidelines,^2^ it is sometimes challenging to do so and requires multiple modalities, including high-dose opioids, palliative radiotherapy (RT), adjuvant analgesics, or nerve block as the underlying pathophysiologies are heterogeneous.^3^ Currently, there are no universal tools for predicting the necessity of high-dose opioid for cancer-related pain. Some pain prognostic scales, such as the Edmonton Pain Staging System or Cancer Pain Prognostic Scale, have been validated.^4,5^ However, patients who are seen in a referral institute (hospice, hospital, or pain clinic) might have a different spectrum of problems or concerns; therefore, these prognostic scales may not be generalized to all patients with advanced cancer.

We attempted to identify factors related to high-dose opioid use for cancer-related pain to find out clinical profiles of the risk patients. In this study, we assumed that high-dose opioid use had a correlation with intractable cancer-related pain. Although high-dose opioid use was not a direct surrogate end point of intractable cancer pain, it was chosen as an end point because it could be more objectively and quantitatively scored than numerical rating scale. We determined a regression equation to predict whether the patient would require a high-dose opioid or not. Identification of clinical factors requiring high-dose opioids in patients with cancer-related pain would be helpful for earlier and better management of cancer-related pain.

## Methods

### Study design and datasets

In-hospital cancer patients who were referred to the palliative care team for cancer pain between April 1, 2014 and July 31, 2019 were eligible for this study. One patient comprised one record, even if the same patients were consulted at different time points. Patients who did not receive interventions for cancer pain were excluded. Data were collected from medical records. Collected data included patient characteristics (age, gender, and performance status [PS] at presentation); type of cancer (clinical departments) requesting consultations; locus of pain; type of pain (somatic, visceral, neuropathic, and/or unknown cause); type of analgesics other than opioids (nonsteroidal anti-inflammatory drugs [NSAIDs] and adjuvant analgesics); type of opioid at induction; the highest dose of oral morphine equivalent, which was calculated according to the conversion table of oral or parenteral opioids; other modalities (palliative RT and nerve block); and opioid switch. Psychological factors (distress, depression, anxiety, or fear) were not assessed because no objective scales were available in the medical records.

Clinical factors significantly correlated to the usage of a high-dose opioid with >120 mg/day of oral morphine equivalent were determined. The definition of high-dose opioid use follows that of “Guidance for Proper Use of Medical Narcotics” published by “Ministry of Health, Labour and Welfare of Japan” and “Guideline for Pharmacologic Management of Neuropathic Pain” by Japan Society of Pain Clinicians.^6,7^ The amount of rescue opioid use was not included.

The study was reviewed and approved by the Ethics Committee of the Shiga University of Medical Science.

### Statistical analysis

Patients were assigned to either of the two groups according to the highest daily oral morphine equivalent dose: high-dose opioid group (>120 mg/day oral morphine equivalent use) and low-dose opioid group (<120 mg/day oral morphine equivalent use). We transformed these two groups into binary data (low-dose opioid group = 0, high-dose opioid group = 1) as dependent variables. A logistic regression analysis was performed to identify factors that were significantly associated with the high-dose opioid group. The independent variables included the following clinical factors: presence or absence of somatic pain, visceral pain, neuropathic pain or unknown cause; the use of adjuvant analgesics, NSAIDs, palliative RT, nerve block, and opioid switch; and gender, age, PS, type of induction opioid, and type of cancer (clinical departments) requesting consultations. The patients were excluded from the analysis if the use of high-dose opioid preceded interventions with adjuvant analgesics, NSAIDs, palliative RT, nerve block, or opioid switch. All variables were transformed into binary data (absence into 0, presence into 1) except age, which is a continuous variable. Independent factors showing a significant correlation that were highly intercorrelated (correlation coefficient, *r* > 0.7) were excluded because of multicollinearity. The ordinal variable, PS, was categorized as follows: R, PS 0 or 1; C1, PS 2; C2, PS 3; C3, PS 4. PS 0 and 1 belonged to one category because the count of PS 0 was too less (*n* = 3). The nominal variables, type of cancer (clinical departments) requesting consultations, were categorized as follows: R, respiratory cancer; C1, urological cancer; C2, gastrointestinal, hepatobiliary, or pancreatic cancer; C3, otolaryngology cancer; C4, breast cancer; C5, dermatological cancer; C6, orthopedic cancer; C7, other cancer (hematological, neurosurgical, pediatric, gynecological, oral, and other). We selected the respiratory cancer as a reference category because it showed the highest percentage of high-dose opioid use. The departments in C7 belonged to one category as their individual numbers were too low to be categorized. The variables with several large missing values (unknown cause and type of opioid at consultation) were excluded. Multivariate logistic regression was used to calculate odds ratios (ORs) and 95% confidence intervals (CIs) after simultaneously controlling for potential confounders. We used IBM SPSS Statistics version 25.

## Results

Table 1 shows the characteristics and demographics of 385 patients referred to the palliative care team due to cancer pain. Several factors, which included the type of pain: others/unknown and type of opioid at the consultation, which had a large amount of missing data (159 and 90, respectively), were removed from the factors for logistic regression analysis.

**Table 1.** Patient Characteristics and Extracted Factors That May Affect High-Dose Opioid Use (*n* = 385)

	*n* (%)	Median	Range
Demographic factors			
Gender (male)	246 (63.9)		
Age		67	11–93
PS			
0	3 (0.8)		
1	94 (24.4)		
2	80 (20.8)		
3	134 (34.8)		
4	74 (19.2)		

	*n* (%)	Available data (*n*)	Missing data (*n*)
Type of pain			
Somatic pain (yes)	211 (54.8)	379	6
Visceral pain (yes)	221 (57.4)	377	8
Neuropathic pain (yes)	101 (26.2)	375	10
Others/unknown (yes)	27 (7.0)	226	159
Type of analgesic therapy			
Adjuvant analgesics (yes)	116 (30.1)	382	3
NSAIDs (yes)	247 (64.2)	381	4
Palliative RT (yes)	88 (22.9)	381	4
Nerve block	11 (2.9)	379	6
Opioid switch (yes)	125 (32.5)	373	12
Type of opioid at consultation			
Morphine (yes)	74 (19.2)	295	90
Oxycodone (yes)	134 (34.8)	295	90
Fentanyl (yes)	87 (22.6)	295	90
Type of cancer			
Orthopedic	17 (4.4)	381	4
Dermatological	16 (4.2)	381	4
Breast	30 (7.8)	381	4
Otolaryngology	30 (7.8)	381	4
Respiratory	69 (17.9)	381	4
G-I, hepatobiliary, pancreatic	139 (36.1)	381	4
Urological	52 (13.5)	381	4
Others	28 (7.3)	381	4

G-I, gastrointestinal; NSAIDs, nonsteroidal anti-inflammatory drugs; PS, performance status; RT, radiotherapy.

We aimed to determine variables significantly related to high-dose opioid use (>120 mg/day oral morphine equivalent use). Patient characteristics of high- and low-dose opioid groups are shown in Table 2. Multicollinearity was confirmed by the correlation coefficient matrix, and no independent factors showed a significant correlation (*r* < 0.7). A logistic regression analysis was conducted; we used a forward stepwise selection method. The model chi-square test was significant (*p* < 0.001). The goodness-of-fit test by Hosmer and Lemeshow showed *p* = 0.292, thereby confirming the fitness. The percentage of correct classifications was 86.9%. Outliers in which predicted values exceeded ±3 standard deviation of measured values did not exist. Table 3 shows the independent variables with OR, 95% CI, and *p*-values. Among the variables, age (OR 0.965, 95% CI 0.944–0.986, *p* = 0.001), respiratory cancers (OR 1.882, 95% CI 1.069–3.312, *p* < 0.001), and opioid switch (OR 2.869, 95% CI 1.497–5.497, *p* = 0.001) were determined to be significant.

**Table 2.** Patient Characteristics Between High-Dose and Low-Dose Opioid Use Groups

	High-dose opioid, *n* (%)	Low-dose opioid, *n* (%)
*N*	52	333
Gender (male)	29 (55.8)	217 (65.2)
Age (median)	59.5 (range: 22–82)	68 (range: 11–93)
PS		
0/1	17 (32.7)	80 (24.0)
2	13 (25.0)	67 (20.1)
3	22 (23.1)	122 (36.6)
4	10 (19.2)	64 (19.2)
Type of pain		
Somatic pain (yes)	31 (59.6)	180 (54.9)
Visceral pain (yes)	30 (57.7)	191 (58.4)
Neuropathic pain (yes)	16 (30.8)	85 (26.2)
Type of analgesic therapy		
Adjuvant analgesics (yes)	22 (42.3)	94 (28.5)
NSAIDs (yes)	43 (82.7)	204 (62.0)
Palliative RT (yes)	16 (30.8)	72 (21.8)
Nerve block	3 (5.8)	8 (2.4)
Opioid switch (yes)	29 (55.8)	96 (29.7)
Type of opioid at consultation		
Morphine (yes)	7 (13.5)	67 (20.6)
Oxycodone (yes)	21 (40.4)	113 (34.7)
Fentanyl (yes)	22 (42.3)	65 (19.9)
Type of cancer		
Orthopedic	4 (7.7)	13 (4.0)
Dermatological	0 (0.0)	16 (4.9)
Breast	1 (1.9)	29 (8.8)
Otolaryngology	3 (5.8)	27 (8.2)
Respiratory	20 (38.5)	49 (14.9)
G-I, hepatobiliary, and pancreatic	16 (30.8)	123 (37.4)
Urological	3 (5.8)	49 (14.9)
Others	5 (9.6)	23 (7.0)

**Table 3.** Logistic Regression Analysis Identifying Factors Related to High-Dose Opioid Use

Variables	Partial regression coefficient	OR (95% CI)	*p*
Age	−0.036	0.965 (0.944–0.986)	0.001
Respiratory cancers	1.282	1.882 (1.069–3.312)	<0.001
Opioid switch	1.054	2.869 (1.497–5.497)	0.001
Constant	−0.428	0.651	

Model chi-square test *p* < 0.001. % of correct classifications 86.9%. Logistic regression equation: Log (p/(1-p)) = −0.036 × [Age] + 1.282 × [Respiratory Dep.] + 1.282 × [Opioid switch] −0.428.

CI, confidence interval; OR, odds ratio.

## Discussion

In this study, we retrospectively analyzed 385 patients with cancer-related pain due to various disease conditions. A logistic regression analysis was performed to determine clinical factors associated with high-dose opioid use (>120 mg/day oral morphine equivalent use), which might be related to intractable cancer pain. Factors associated with the need for high-dose opioids were determined to be younger age, respiratory cancers, and use of opioid switching. Of these, the most influential factor was opioid switching (OR 2.869, 95% CI 1.497–5.497). Several previous studies have shown that aged cancer patients need lower doses of opioids than younger patients.^8–10^ In addition, some earlier studies suggest that women are more sensitive to pain.^11,12^ However, in our analysis, gender was not a significant associated factor. To the best of our knowledge, the use of opioid switch might not have been identified as an independent associated factor for high-dose opioid use in the literature. This factor indicates the need for multimodal interventions for cancer-related pain.

We recognize that each hospital might have a different spectrum of problems or concerns. Therefore, our retrospective analysis may have a selection bias and limitations. Although the rate of correct classification was relatively high (86.9%), we must be careful regarding the generality of this equation. We chose several clinical factors as variables; however, some potentially important factors, such as psychological, social, or spiritual matters, could not be included because of the lack of information about their objective assessment from the retrospective medical records. It is well known that cancer pain has been associated with psychosocial factors such as psychological distress and emotional and spiritual factors.^13–17^ A future prospective study using an objective scale, such as the Functional Assessment of Cancer Therapy^18^ or Mental Health Inventory^19^ might clarify the significance of these factors.

In conclusion, we conducted a statistical analysis to identify factors associated with the requirement of high-dose opioids in palliative patients with cancer-related pain. Our study indicates that younger age, respiratory cancers, and opioid switch are significantly related to the necessity for high-dose opioid use. Within the limitations of the study, the statistical identification of factors associated with high-dose opioid use might contribute to the establishment of evidence-based medicine in pain relief and palliative care.

## Abbreviations Used

CI: confidence interval
G-I: gastrointestinal
NSAIDs: nonsteroidal anti-inflammatory drugs
OR: odds ratio
PS: performance status
RT: radiotherapy
